# Direct admission to the intensive care unit from the emergency department and mortality in critically ill hematology patients

**DOI:** 10.1186/s13613-019-0587-7

**Published:** 2019-10-02

**Authors:** Olivier Peyrony, Sylvie Chevret, Anne-Pascale Meert, Pierre Perez, Achille Kouatchet, Frédéric Pène, Djamel Mokart, Virginie Lemiale, Alexandre Demoule, Martine Nyunga, Fabrice Bruneel, Christine Lebert, Dominique Benoit, Adrien Mirouse, Elie Azoulay

**Affiliations:** 1 Emergency Department, Hôpital Saint-Louis, 1 avenue Claude Vellefaux, 75010 Paris, France; 2 Biostatistics and Medical Information Department, Hôpital Saint-Louis, Paris, France; 3 Centre de Recherche en Épidémiologie et Statistiques – Université de Paris (CRESS-INSERM-UMR1153), Epidemiology and Clinical Statistics for Tumor, Respiratory, and Resuscitation Assessments (ECSTRRA) Team, Paris, France; 4 Université de Paris, Paris, France; 5 Intensive Care Unit, Institut Jules Bordet, Université libre de Bruxelles (ULB), Brussels, Belgium; 6 Intensive Care Unit, Hôpital Brabois, Vandoeuvre Les Nancy, France; 7 Intensive Care Unit, Centre hospitalier régional universitaire, Angers, France; 8 Intensive Care Unit, Hôpital Cochin, Paris, France; 9 Institut Cochin, INSERM U1016, CNRS UMR 8104, Paris, France; 10 Intensive Care Unit, Institut Paoli Calmettes, Marseille, France; 11 Intensive Care Unit, Hôpital Saint-Louis, Paris, France; 12 Intensive Care Unit, Hôpital Pitié-Salpêtrière, Paris, France; 13 INSERM, UMRS 1158 Neurophysiologie respiratoire expérimentale et clinique, Paris, France; 14 Université Paris Sorbonne, Paris, France; 15 Intensive Care Unit, Hôpital Victor Provo, Roubaix, France; 16 Intensive Care Unit, Hôpital André Mignot, Versailles, France; 17 Intensive Care Unit, Centre hospitalier départemental Vendee, La Roche Sur Yon, France; 18 Intensive Care Unit, Hôpital universitaire de Ghent, Ghent, Belgium

**Keywords:** Emergency department, Direct admission, Intensive care unit, Hematological malignancy

## Abstract

**Background:**

The aim of this study was to assess the benefit of direct ICU admission from the emergency department (ED) compared to admission from wards, in patients with hematological malignancies requiring critical care.

**Methods:**

Post hoc analysis derived from a prospective, multicenter cohort study of 1011 critically ill adult patients with hematologic malignancies admitted to 17 ICU in Belgium and France from January 2010 to May 2011. The variable of interest was a direct ICU admission from the ED and the outcome was in-hospital mortality. The association between the variable of interest and the outcome was assessed by multivariable logistic regression after multiple imputation of missing data. Several sensitivity analyses were performed: complete case analysis, propensity score matching and multivariable Cox proportional-hazards analysis of 90-day survival.

**Results:**

Direct ICU admission from the ED occurred in 266 (26.4%) cases, 84 of whom (31.6%) died in the hospital versus 311/742 (41.9%) in those who did not. After adjustment, direct ICU admission from the ED was associated with a decreased in-hospital mortality (adjusted OR: 0.63; 95% CI 0.45–0.88). This was confirmed in the complete cases analysis (adjusted OR: 0.64; 95% CI 0.45–0.92) as well as in terms of hazard of death within the 90 days after admission (adjusted HR: 0.77; 95% CI 0.60–0.99). By contrast, in the propensity score-matched sample of 402 patients, direct admission was not associated with in-hospital mortality (adjusted OR: 0.92; 95% CI 0.84–1.01).

**Conclusions:**

In this study, patients with hematological malignancies admitted to the ICU were more likely to be alive at hospital discharge if they were directly admitted from the ED rather than from the wards. Assessment of early predictors of poor outcome in cancer patients admitted to the ED is crucial so as to allow early referral to the ICU and avoid delays in treatment initiation and mis-orientation.

## Background

One of the goals of emergency physicians is to detect, based on clinical characteristics and physiological derangements, patients without established organ dysfunction, but who are at high risk of early life-threatening complications. Such patients should be early admitted to the intensive care unit (ICU) or high dependency units as to benefit from close monitoring. Indeed, when these patients are admitted to classic wards, the level of monitoring may be inappropriate. Moreover, the workload that these high-risk patients impose to clinicians also hampers optimal management of remaining patients. The occurrence of complications in wards could lead to delays in patient optimal care and transfer to the ICU and, therefore, increase morbi-mortality [1]. This is even more likely for immunocompromised patients in whom the risk of deterioration is higher and less easily predictable [2].

Thus, we hypothesized that immunocompromised patients admitted to the ICU directly from the emergency department (ED), were admitted sooner and, may have better outcomes compared to similar patients, admitted to ICU from the wards.

The aim of this study was to assess the benefit of direct ICU admission from the ED compared to admission from wards, in patients with hematological malignancies requiring critical care.

## Methods

This is a post hoc analysis derived from a prospective, multicenter cohort study of 1011 critically ill adult patients with hematologic malignancies admitted to 17 ICUs in Belgium and France from January 1, 2010 to May 1, 2011 [3]. Briefly, the study was carried out in university or university-affiliated centers in France and Belgium that belonged to a research network instituted in 2005. In all 17 centers, a senior intensivist and a senior hematologist are available around the clock and make ICU-admission decisions together. During the study period, consecutive patients having hematologic malignancies who were admitted to the participating ICUs for any reason were included. Exclusion criteria were complete cure of the malignancy for more than 5 years, ICU admission only to maximize safety of a procedure, and age younger than 18 years. For this post hoc analysis, patients with missing data on the outcome were also excluded. The study was approved by the appropriate ethics committees in France and Belgium. All patients or relatives were informed and consented to participate in the study.

### Data collection and outcome measure

Data abstracted from the study database were: age, sex, underlying malignancy, disease status (newly diagnosed if malignancy was diagnosed in the last month, complete or partial remission, other), autologous or allogeneic bone marrow or hematopoietic stem-cell transplantation (BMT/HSCT), treatment with long-course corticosteroids, Charlson comorbidity index, performance status, existence of organ failure based on the Sequential-related Organ Failure Assessment (SOFA) criteria, SOFA score, main reason for ICU admission, circumstances of ICU admission, time between hospital and ICU first request, and between first request and ICU admission, number of requests before ICU admission, specialty and experience of the physician that requested ICU admission, direct admission from the ED, length of ICU and hospital stay, vital status at ICU, at hospital discharge, and at day 90.

The variable of interest was direct admission from the ED, and the main outcome was in-hospital mortality.

### Statistical analysis

Results are reported as medians with interquartile ranges (IQR) for continuous variables and numbers with percentages for binary and categorical variables.

Patient characteristics were compared using the Chi-square test for categorical variables and the Wilcoxon rank sum test or the Student *t* test, as appropriate, for continuous variables (with normality tested with Shapiro–Wilk test). We investigated the association between the variable of interest and the outcome by multivariable logistic regression to search for potential confounders. Characteristics associated with the outcome on the basis of *P*-values less than 0.1 by univariable analyses were included in a multivariable logistic model; clinically relevant prognostic characteristics such as SOFA score, Charlson risk index or performance status, were forced in the model regardless of their *P* value. Then, a backward selection procedure was applied, except for clinically relevant prognostic variables that were not removed from the model.

Missing data were managed with multiple imputation by chained equations [4]. The distribution of the data according to the presence or absence of missing data was checked (plots if continuous or tables if categorical variables) to ensure that missing data were missing completely at random. As recommended [5], variables included in the imputation model were those of the logistic regression prediction model (including the outcome), in addition to auxiliary covariates correlated with the missing variables (i.e., sex, underlying disease, days since diagnosis, days between first call to intensivist and ICU admission, experience of the physician requesting ICU, number of calls before ICU admission, organ failures). Five datasets were imputed with 50 iterations each. Multivariable logistic regression model was applied to the 5 imputed datasets and final estimates were obtained by averaging the 5 estimates according to Rubin’s rules.

Several sensitivity analyses were performed. First, a complete cases analysis was performed. Second, to handle potential residual confounding by indication, a propensity score matching was done, where propensity score of being directly ICU admitted was estimated from a multivariable logistic model, with resulting balances in confounders checked by standardized mean differences and c-index [6], then matching performed without replacement within a caliper of 0.2 standard deviation of the logit of propensity score [7]. Third, we plotted survival Kaplan–Meier curve (from ICU admission to 90 days) according to direct admission status from the ED. Hazards ratio (HR) from Cox proportional-hazards models was used to quantify the association between the direct admission status and the outcome, adjusting for baseline predictors of survival. Underlying assumptions of the Cox model were checked: Proportional hazards (PH) assumption was tested using a formal test based on the Schoenfeld residuals, with time-dependent effect considered for covariates that violated the PH assumption [8]. Log-linearity between non-binary covariates and hazard was assessed through splines; in case of nonlinear effect, covariates were dichotomized according to thresholds derived from the splines.

All *P*-values were two-sided, with values of 0.05 or less considered as statistically significant.

Data were analyzed with R 3.5.0 software (the R Foundation for Statistical Computing, Vienna, Austria).

## Results

### General characteristics

During the study period, 1011 patients were enrolled. Due to missing data on the outcome, 3 patients were excluded leading to a number of 1008 included patients in the analysis. Table 1 shows the patients’ characteristics at ICU admission.

**Table 1:** Patients’ characteristics at ICU admission

Characteristics	Overall cohort (*N* = 1008)	Status at hospital discharge		Missing data
		Alive (*n* = 613)	Death (*n* = 395)	
Age, median [IQR], years	60 [49–69]	59 [47–68]	62 [52–71]	0
Female gender,* n* (%)	396 (39.3)	252 (41.1)	144 (36.4)	0
Underlying malignancy,* n* (%)				0
Non-Hodgkin’s lymphoma	319 (31.6)	190 (31.0)	129 (32.7)	
Acute myeloid leukemia	274 (27.2)	162 (26.4)	112 (28.4)	
Myeloma	126 (12.5)	86 (14.0)	40 (10.1)	
Chronic lymphocytic leukemia	75 (7.4)	45 (7.3)	30 (7.6)	
Acute lymphocytic leukemia	73 (7.2)	44 (7.2)	29 (7.3)	
Myelodysplastic syndrome	46 (4.6)	26 (4.2)	20 (5.1)	
Hodgkin’s disease	25 (2.5)	18 (2.9)	7 (1.8)	
Chronic myeloid leukemia	19 (1.9)	14 (2.3)	5 (1.3)	
Other	51 (5.1)	28 (4.5)	23 (5.8)	
Disease status,* n* (%)				54
Complete or partial remission	232 (24.3)	154 (26.6)	78 (20.7)	
Newly diagnosed	382 (40.0)	238 (41.2)	144 (38.2)	
Other	340 (35.6)	185 (32.1)	155 (41.1)	
Days since diagnosis, median [IQR]	169 [7–965]	163 [7–1015]	174 [10–942]	97
Allogeneic BMT/HSCT recipient,* n* (%)	146 (14.5)	70 (11.4)	76 (19.3)	3
Long course corticosteroids, *n* (%)	381 (38)	218 (35.7)	163 (41.6)	5
Charlson comorbidity index, median [IQR]	4 [3–6]	4 [2–5]	4 [3–6]	1
Poor PS (> 2),* n* (%)	198 (19.8)	85 (13.9)	113 (28.8)	6
Reason for ICU admission, *n* (%)				55
Acute respiratory failure	374 (39.2)	205 (35.6)	169 (44.8)	
Sepsis or septic shock	255 (26.8)	166 (28.8)	89 (23.6)	
Metabolic disorder or acute kidney injury	111 (11.6)	73 (12.7)	38 (10.1)	
Coma	69 (7.2)	41 (7.1)	28 (7.4)	
Other	144 (15.5)	91 (15.8)	53 (14.1)	
Organ failure, *n* (%)				2
Respiratory	631 (62.7)	344 (56.1)	287 (73.0)	
Hemodynamic	426 (42.3)	236 (38.5)	190 (48.3)	
Renal	305 (30.3)	171 (27.9)	134 (34.0)	
Neurological	226 (22.4)	114 (18.6)	112 (28.4)	
Coagulation	194 (19.3)	99 (16.1)	95 (24.2)	
Hepatic	83 (8.3)	39 (6.4)	44 (11.2)	
Multi-organ	549 (54.5)	289 (47.1)	260 (66.0)	
SOFA score, median [IQR]	6 [3–9]	5 [3–7]	7 [5–11]	1

*BMT* bone marrow transplantation, *HSCT* hematopoietic stem-cell transplantation, *ICU* intensive care unit, *IQR* interquartile range, *PS* performance status, *SOFA* Sequential Related Organ Failure Assessment

Patients were mostly men (60.7%) and half were aged of less than 60 years. The most frequent malignancy was non-Hodgkin’s lymphoma (31.6%), followed by acute myeloid leukemia (27.2%) and myeloma (12.5%). Chronic malignancy as chronic lymphocytic or myeloid leukemia or myelodysplastic syndrome was present in 13.9% of the patients. Disease status was complete or partial remission or newly diagnosed for 64.3% of the patients. One hundred and ninety-eight patients (19.8%) had poor performance status. The most frequent main reasons for ICU admission were acute respiratory failure (39.2%) and sepsis (26.8%). Patients had respiratory failure in 62.7% cases, cardiovascular failure in 42.3% cases, renal failure in 30.3% cases, and neurological failure in 22.4% cases.

### Direct ICU admission from the ED and hospital mortality

**Fig. 1:**
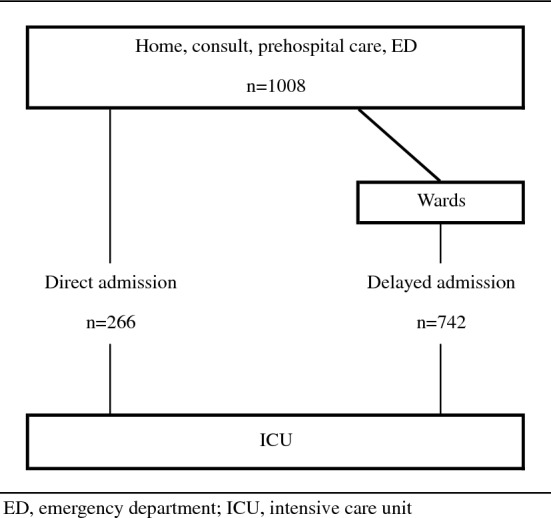
Flowchart of patients depending on their modalities of admission to ICU

Table 2 shows the modalities of ICU admission. ICU admission was requested by the hematologist in 59.3% of the cases and by an emergency physician in 22% of the cases. Physicians requested ICU transfer in the 4 [0–17] days after patient hospital admission. Patients were admitted to ICU most of the time by the day of the request (97.6%) and after one call to the intensivist (87.7%). Direct ICU admission from the ED occurred for 266 (26.4%) patients (Fig. 1). Patients stayed in ICU 5 [2–11] days in median. ICU and in-hospital mortality were 27.7% and 39.2%, respectively.

**Table 2:** Modalities of ICU admission and hospital mortality

Characteristics	Overall cohort (*N* = 1008)	Status at hospital discharge		Missing data
		Alive (*n* = 613)	Dead (*n* = 395)	
Days between hospitalization and first call to intensivist, median [IQR]	4 [0–17]	2 [0–14]	7 [1–21]	44
Days between first call to intensivist and ICU admission, median [IQR]	0 [0–0]	0 [0–0]	0 [0–0]	5
ICU admission requested by,* n* (%)				12
Emergency physician	219 (22.0)	150 (24.7)	69 (17.7)	
Other	777 (78.0)	456 (75.2)	321 (82.3)	
Experience of the physician requesting ICU,* n* (%)				25
Senior physician	651 (66.2)	387 (64.3)	264 (69.3)	
Fellow	170 (17.3)	112 (18.6)	58 (15.2)	
Resident/intern	162 (16.5)	103 (17.1)	59 (15.5)	
Number of calls before ICU admission, *n* (%)				151
1	752 (87.7)	474 (88.9)	278 (85.8)	
≥ 2	105 (12.3)	59 (11.1)	46 (14.2)	
Direct ICU admission from the ED, *n* (%)	266 (26.4)	182 (29.7)	84 (21.3)	0
ICU length of stay, median [IQR], days	5 [2–11]	5 [2–9]	5 [2–13]	0
Hospital length of stay, median [IQR], days	28 [13–47]	29 [16–47]	25 [9–47]	39
Death at ICU discharge,* n* (%)	279 (27.7)	0 (0)	279 (70.6)	0

*ED* emergency department, *ICU* intensive care unit, *IQR* interquartile range

By univariable analyses (Additional file 1), variables associated with in-hospital mortality were an age > 60 (44% vs. 34.6%; *P *= 0.003), non-complete or non-partial remission or non-newly diagnosed disease status (45.6% vs. 36.2%; *P *= 0.005), allogeneic BMT/HSCT recipient (52.1% vs. 36.9%; *P *< 0.0001), a high Charlson comorbidity index (median: 4; IQR: 3 to 6 *vs* median: 4; IQR: 2 to 5; *P *= 0.0001), a poor performance status (57.1% vs. 34.7%; *P *< 0.0001), a high SOFA score (median: 7; IQR 5 to 11 *vs* median: 5; IQR 3 to 7; *P *< 0.0001), time between hospitalization and first call to the intensivist (median: 7; IQR 1 to 21 *vs* median: 2; IQR 0 to 14; *P *< 0.0001), an ICU admission requested by a non-emergency physician (41.3% vs. 31.5%; *P *= 0.01). Conversely, direct ICU admission from the ED was associated with decreased in-hospital mortality (31.6% vs. 41.9%; *P *= 0.004). Table 3 reports the estimated effect of direct ICU admission on the in-hospital mortality adjusted on prognostic variables, based on the multivariable logistic model after multiple imputation. Direct ICU admission from the ED was associated with decreased in-hospital mortality (OR: 0.63; 95% CI 0.45 to 0.88).

**Table 3:** Multivariable analysis. Variables independently associated with hospital mortality

Variables	Model without imputation (*N* = 898)			Model with imputation (*N* = 1008)		
	OR	95% CI	*P*	OR	95% CI	*P*
Direct admission to the ICU from the ED	0.64	(0.45 to 0.92)	0.02	0.63	(0.45 to 0.88)	0.007
Age > 60 years	1.47	(1.04 to 2.10)	0.03	1.47	(1.05 to 2.04)	0.02
Disease status						
Remission or newly diagnosed	1.00					
Other	1.49	(1.08 to 2.06)	0.01	1.52	(1.12 to 2.07)	0.008
Allogeneic BMT/HSCT recipient	2.46	(1.57 to 3.86)	< 0.0001	2.42	(1.58 to 3.71)	< 0.0001
Charlson (/point)	1.06	(0.99 to 1.14)	0.10	1.07	(1.00 to 1.15)	0.04
Poor PS (> 2)	1.88	(1.30 to 2.72)	< 0.001	1.99	(1.40 to 2.83)	0.0001
SOFA score (/point)	1.24	(1.19 to 1.29)	< 0.00001	1.23	(1.19 to 1.28)	< 0.00001
Reason for ICU admission						
Sepsis or septic shock	1.00					
Acute respiratory failure	2.16	(1.47 to 3.2)	< 0.001	2.11	(1.45 to 3.06)	< 0.0001
Coma	1.68	(0.89 to 3.15)	0.10	1.72	(0.94 to 3.15)	0.08
Metabolic disorder or acute kidney injury	2.05	(1.17 to 3.56)	0.01	2.12	(1.24 to 3.62)	0.006
Other	2.17	(1.30 to 3.63)	0.003	2.25	(1.38 to 3.67)	0.001

*BMT* bone marrow transplantation, *ED* emergency department, *HSCT* hematopoietic stem-cell transplantation, *ICU* intensive care unit, *PS* performance status, *SOFA* Sequential-Related Organ Failure Assessment

### Sensitivity analyses

**Fig. 2:**
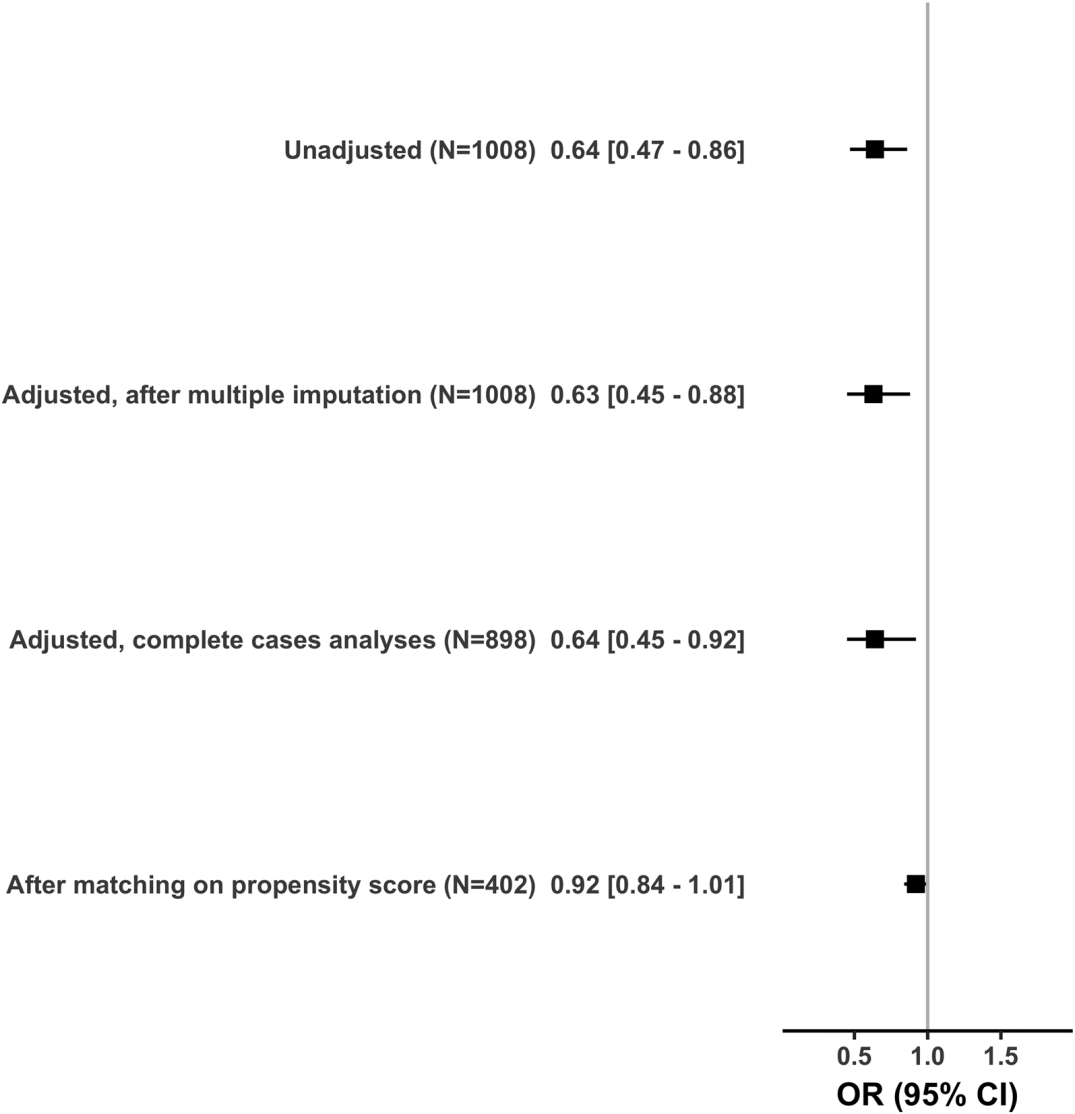
Effects of direct ICU admission from the ED on hospital mortality

Figure 2 summarizes the estimated effects of direct ICU admission from the ED on in-hospital mortality depending on the sensitivity analysis.

First, the complete cases analysis confirmed the improved survival of direct ICU admission (OR: 0.64; 95% CI 0.45–0.92).

Secondly, based on propensity score, 201 patients admitted to the ICU directly from the ED were matched with patients admitted from wards. Additional file 2 reports differences in patient characteristics according to location prior to ICU admission before matching and Additional file 3 shows the absolute mean differences in patient characteristics before and after matching on propensity score. In the matched sample of 402 patients, direct admission was not associated with in-hospital mortality (OR: 0.92; 95% CI 0.84–1.01).

**Fig. 3:**
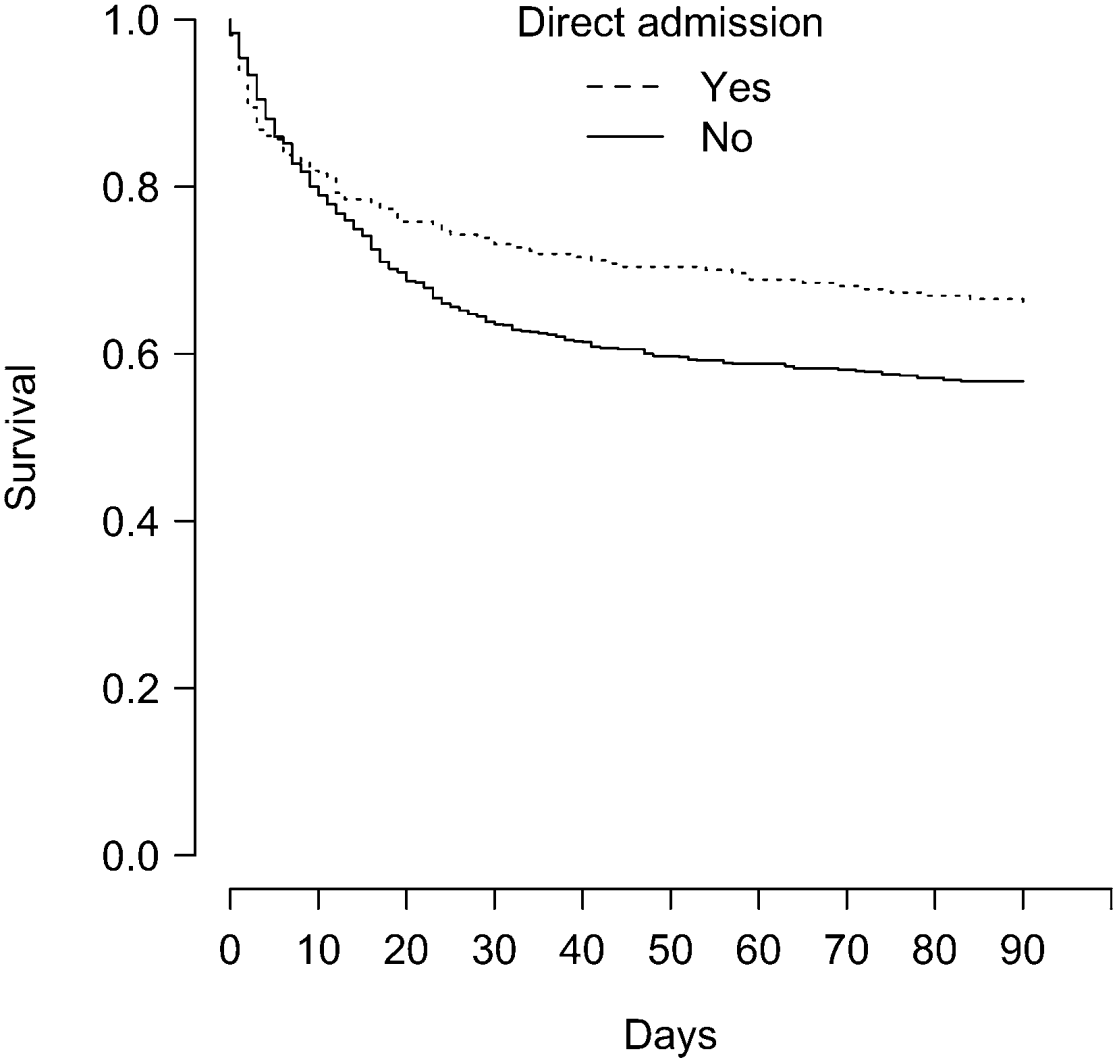
Kaplan–Meier survival during 90 days from intensive care unit admission depending on direct admission from the emergency department

Last, survival was significantly improved in patients who were admitted directly to the ICU from the ED (Fig. 3), with an estimated unadjusted HR at 0.75 (95% CI 0.59–0.94, *P *= 0.014). After adjustment on survival predictors (namely, disease status, BMT/HSCT, Charlson comorbidity index, performance status and SOFA score at ICU admission), the adjusted HR was 0.77 (95% CI 0.60 to 0.99, *P *= 0.04).

## Discussion

In this post hoc analysis derived from a prospective, multicenter cohort study of 1011 critically ill adults patients with hematologic malignancies admitted to ICU, direct admission from the ED was associated with decreased in-hospital mortality in comparison with admission from the wards. This effect on mortality was confirmed in most of the sensitivity analyses except after matching on propensity score.

Benefit from appropriate and prompt ICU management is an established fact in myocardial infarction [9], ischemic stroke [10] or major trauma [11]. Along this line, early ICU admission of patients with some physiological derangement, before organ dysfunction actually develops, may be beneficial. Chalfin et al. showed that among 50,322 patients admitted to the ICU from the ED, those admitted with a ≥ 6 h delay had higher increased hospital length of stay (7 vs. 6 days, *P *< 0.001) and higher ICU and hospital mortality rates (10.7% vs. 8.4%, *P* < 0.01 and 17.4% vs. 12.9%, respectively, *P* < 0.001) [12]. This is also in agreement with the Renaud et al. study where patients with community-acquired pneumonia had different survival according to whether they were admitted to the ICU early, late or not admitted to the ICU (adjusted OR 2.63; 95% CI 1.42–4.90 for patients admitted early to the ICU) [13].

The use of ICU resources needs to be carefully discussed, however. Bed availability remains a major determinant of outcomes in settings where ICU resources are scarce [14]. Similarly, Cardoso et al. [15] found that delayed admission of patients requiring ICU management but for whom no bed was available had worse outcome than patients admitted immediately. They found that each waiting hour was independently associated with a 1% increase in risk of hospital death (hazard ratio = 1.010; 95% CI 1.002 to 1.018).

Patients with subtle physiological derangements that could foreshadow organ failure must be close monitored so as to prevent secondary deterioration. Hence, it may be hazardous to have these patients transferred to the wards. In a cohort of 841 septic patients transferred from the ED to the wards, Whittaker et al. [16] reported that 12.5% of patients were transferred to the ICU within 48 h and/or died within 28 days of admission. In this study, cancer was an independent risk factor of adverse outcome. Other studies have shown that septic patients admitted to ICU from wards had higher mortality rates [17, 18]. Last, in a study of cancer patients for whom an ICU admission was requested, 20% of the patients transferred to the wards because they were “too well” died [19]. This study suggests that ICU admission might have been inappropriately delayed and that delayed admission is associated with a high mortality rate.

Benefits from early ICU admission have also been reported in high-risk patients with malignancies and acute respiratory failure. In a prospective study on 219 patients with cancer admitted to the ICU for acute respiratory failure in France conducted by Mokart et al. [20], time between respiratory symptoms onset and ICU admission of at least 2 days was an independent predictor of day-28 mortality (OR: 2.65; 95% CI 1.29–5.44). In the present study, neither the delay between ICU request and ICU admission nor the number of calls before ICU admission was associated with in-hospital mortality by univariable analyses. These two variables were not included in the logistic regression model because of their colinearity with our variable of interest. Early detection of physiological derangement to allow prompt ICU admission is crucial and may be compromised in wards where surveillance is not optimal. Dedicated emergency teams have shown to be beneficial in case of soon intervention. After implementation of a medical emergency team in a comprehensive cancer center in Seoul [21], Song et al. [2] showed that in-hospital mortality rates increased significantly with increasing quartiles of time from physiological derangement to medical emergency team intervention in 199 critically ill cancer patients admitted to the ICU. Late intervention after 1.5 h after physiological derangement was significantly associated with in-hospital mortality in the multivariable analysis with adjustment for propensity score (OR: 3.91; 95% CI 1.93 to 7.94). These results remained true for long-term mortality in a study including 525 patients with cancer where early intervention was significantly associated with 1-year mortality after adjusting for potential confounders (hazard ratio: 0.456; 95% CI 0.348 to 0.597) [22].

Deciding the accurate timing for ICU admission may be challenging. ICU referral or admission decisions may be even more difficult in cancer patient when prognosis is uncertain [23]. Studies to incite early ICU management in high-risk patients are warranted. Besides survival benefits, organ dysfunction recovery and ability to receive optimal cancer care are critical endpoints of such interventions.

## Limitations

Our study has several limitations. First, this was a post hoc analysis of a cohort study dealing with the comparison of non-randomized groups. Obviously, because randomizing modalities of ICU admission may be complex, baseline characteristics of patients admitted from wards differ from those admitted directly from the ED. To handle these differences and limit selection bias, we performed a multivariable logistic regression to incorporate differences in baseline characteristics of these groups; however, it is possible that non-observed confounding factors exist such as hospital bed availability. Secondly, estimated benefit disappeared in the propensity score-matched sample, but this may be at least partially due to a lack of power given the limited sample size (402 vs. 1008) and on differences between matched and unmatched patients resulting in a selected population. However, variables that differed between both groups were already part of the multivariable logistic regression, except the type of underlying malignancy and the use of long-term corticosteroids. Thus, we reran a second multivariable analysis including these two variables and direct ICU admission remained associated with a better outcome. Third, we focus our research on patients with hematological malignancies admitted to the ICU and did not consider patients admitted to the wards who did not require or who were not referred to the ICU. Furthermore, patients admitted to the ICU from wards were not necessarily managed in the ED before. Fourth, we could not assess the time elapsed between the need for and the actual ICU admission. It could have been interesting to determine if patients admitted in wards had longer delays between first symptoms and ICU admission reflecting flaws in monitoring. Fifth, we did not consider the outcomes after hospital discharge. It is possible that a substantial part of these patients was transferred to palliative care unit lowering the benefit of survival at discharge.

## Conclusions

In summary, in this post hoc study, patients with hematological malignancies admitted to the ICU were more likely to be alive at hospital discharge if they were directly admitted from the ED rather than from the wards. We are not yet to pretend that patients admitted to the ICU from wards who were previously managed in the ED should have been directly admitted from there. We, however, put forward that these high-risk patients need prompt specific management and close monitoring when they develop subtle physiological derangement to anticipate organ dysfunctions. This close monitoring may be inadequate in the wards and should be achieved by specialized teams in intensive care or high dependency units. Assessment of alarm signs and early predictors of poor outcome in cancer patients admitted to the ED is crucial so as to allow early referral to the ICU and avoid delays in treatment initiation and mis-orientation.

## Supplementary information

**Additional file 1.** Univariable analysis. Variables associated with hospital mortality. *BMT* bone marrow transplantation, *CLL* chronic lymphocytic leukemia, *CML* chronic myeloid leukemia, *ED* emergency department, *HSCT* hematopoietic stem-cell transplantation, *ICU* intensive care unit, *MDS* myelodysplastic syndrome, *PS* performance status, *SOFA* Sequential Related Organ Failure Assessment.

**Additional file 2.** Comparison of patient characteristics according to direct ICU admission. *ALL* acute lymphocytic leukemia, *AML* acute myeloid leukemia, *BMT* bone marrow transplantation, *CLL* chronic lymphocytic leukemia, *CML* chronic myeloid leukemia, *ED* emergency department, *HSCT* hematopoietic stem-cell transplantation, *ICU* intensive care unit, *IQR* interquartile range, *MDS* myelodysplastic syndrome, *PS* performance status, *SOFA* Sequential Related Organ Failure Assessment.

**Additional file 3.** Loveplot. Absolute mean differences in patient characteristics before (unadjusted) and after (adjusted) matching on propensity score. *BMT* bone marrow transplantation, *CLL* chronic lymphocytic leukemia, *CML* chronic myeloid leukemia, *HSCT* hematopoietic stem-cell transplantation, *ICU* intensive care unit, *MDS* myelodysplastic syndrome, *PS* performance status, *SOFA* Sequential Related Organ Failure Assessment.

## Data Availability

The datasets generated and analyzed during the current study are not publicly available due to the potential risk of leakage of personally identifiable information

## Abbreviations

ALL: acute lymphocytic leukemia
AML: acute myeloid leukemia
BMT: bone marrow transplantation
CLL: chronic lymphocytic leukemia
CML: chronic myeloid leukemia
ED: emergency department
HSCT: hematopoietic stem-cell transplantation
ICU: intensive care unit
IQR: interquartile range
MDS: myelodysplastic syndrome
PS: performance status
SOFA: Sequential-related Organ Failure Assessment
