# Supplementary material for: Direct admission to the intensive care unit from the emergency department and mortality in critically ill hematology patients
Source: Ann Intensive Care. 2019 Oct 2;9:110. doi: 10.1186/s13613-019-0587-7 (PMC6775178; doi:10.1186/s13613-019-0587-7)
Supplement: Supplementary file 1 — Additional file 1. Univariable analysis. Variables associated with hospital mortality. BMT bone marrow transplantation, CLL chronic lymphocytic leukemia, CML chronic myeloid leukemia, ED emergency department, HSCT hematopoietic stem-cell transplantation, ICU intensive care unit, MDS myelodysplastic syndrome, PS performance status, SOFA Sequential Related Organ Failure Assessment. [file 13613_2019_587_MOESM1_ESM.pdf]

Additional file 1: Univariable analysis. Variables associated with hospital mortality

Variable	OR	95% CI	*P*	Missing data
Age > 60 years	1.48	(1.15 to 1.91)	0.003	0
Male gender	1.22	(0.94 to 1.58)	0.16	0
Underlying malignancy			0.41	0
Acute leukemia	1			
Chronic malignancy (CLL, CML, MDS)	0.95	(0.63 to 1.41)		
Lymphoma	0.96	(0.70 to 1.30)		
Myeloma	0.68	(0.44 to 1.04)		
Other	1.20	(0.66 to 2.17)		
Disease status			0.005	54
Complete or partial remission or newly diagnosed	1			
Other	1.48	(1.12 to 1.94)		
Days since diagnosis	1.00/day	(0.99 to 1.01)	0.99	97
Allogeneic BMT/HSCT recipient	1.86	(1.30 to 2.65)	0.0007	3
Long course corticosteroids	1.28	(0.99 to 1.66)	0.07	5
Charlson comorbidity index	1.10/point	(1.04 to 1.15)	0.0001	1
Poor PS (>2)	2.50	(1.83 to 3.44)	<0.00001	6
Reason for ICU admission			0.06	55
Sepsis or septic shock	1			
Acute respiratory failure	1.54	(1.11 to 2.14)		
Coma	1.27	(0.73 to 2.19)		
Metabolic disorder or acute kidney injury	0.97	(0.60 to 1.55)		
Other	1.09	(0.71 to 1.66)		
SOFA score	1.22/point	(1.18 to 1.26)	<0.00001	1
Days between hospitalization and first call to intensivist	1.01/day	(1.01 to 1.02)	<0.00001	44
Days between first call to intensivist and ICU admission	1.00/day	(0.93 to 1.07)	0.31	5
ICU admission requested by emergency physician	0.65	(0.47 to 0.90)	0.01	12
Experience of the physician requesting ICU			0.25	25
Senior physician	1			
Fellow	0.76	(0.53 to 1.08)		
Resident/intern	0.84	(0.59 to 1.20)		
Number of calls before ICU admission ≥ 2	1.33	(0.88 to 2.01)	0.21	151
Direct admission from the ED to the ICU	0.64	(0.47 to 0.86)	0.004	0

BMT bone marrow transplantation, *CLL* chronic lymphocytic leukemia, *CML* chronic myeloid leukemia, *ED* emergency department, *HSCT* hematopoietic stem-cell transplantation, *ICU* intensive care unit, *MDS* myelodysplastic syndrome, *PS* performance status, *SOFA* Sequential Related Organ Failure Assessment
