# Supplementary material for: Direct admission to the intensive care unit from the emergency department and mortality in critically ill hematology patients
Source: Ann Intensive Care. 2019 Oct 2;9:110. doi: 10.1186/s13613-019-0587-7 (PMC6775178; doi:10.1186/s13613-019-0587-7)
Supplement: Supplementary file 2 — Additional file 2. Comparison of patient characteristics according to direct ICU admission. ALL acute lymphocytic leukemia, AML acute myeloid leukemia, BMT bone marrow transplantation, CLL chronic lymphocytic leukemia, CML chronic myeloid leukemia, ED emergency department, HSCT hematopoietic stem-cell transplantation, ICU intensive care unit, IQR interquartile range, MDS myelodysplastic syndrome, PS performance status, SOFA Sequential Related Organ Failure Assessment. [file 13613_2019_587_MOESM2_ESM.pdf]

Additional file 2: Comparison of patient characteristics according to direct ICU admission

	Direct ICU admission from the ED	
	No (n=742)	Yes (n=266)
Age > 60 years, n (%)	346 (46.6)	145 (54.5)
Female gender, n (%)	291 (39.2)	105 (39.5)
Underlying malignancy, n (%)		
ALL - AML	284 (38.3)	63 (23.7)
CLL - CML - MDS	85 (11.5)	55 (20.7)
Lymphoma - Hodgkin's disease	246 (33.2)	98 (36.8)
Myeloma	90 (12.1)	36 (13.5)
Other	37 (5.0)	14 (5.3)
Disease status, n (%)		
Remission or newly diagnosed	468 (66.4)	146 (58.6)
Other	237 (33.6)	103 (41.4)
Days since diagnosis, median [IQR]	139 [9-760]	290 [1-1,651]
Allogeneic BMT/HSCT recipient, n (%)	121 (16.4)	25 (9.4)
Long course corticosteroids, n (%)	287 (38.9)	94 (35.5)
Charlson comorbidity index, median [IQR]	4 [3-5]	4 [3-6]
Poor PS (> 2), n (%)	164 (22.2)	34 (12.9)
Reason for ICU admission, n (%)		
Sepsis or septic shock	170 (24.3)	85 (33.7)
Acute respiratory failure	295 (42.1)	79 (31.3)
Coma	53 (7.6)	16 (6.3)
Metabolic disorder or acute kidney injury	75 (10.7)	36 (14.3)

Other	108 (15.4)	36 (14.3)
SOFA score, median [IQR]	6 [3-9]	6 [3-8]
Ventilation, n (%)	460 (62.0)	147 (55.3)
Mechanical ventilation	363 (48.9)	117 (44.0)
Non invasive ventilation	237 (31.9)	65 (24.4)
Amine use, n (%)	387 (52.2)	121 (45.5)
Renal replacement therapy, n (%)	205 (27.6)	66 (24.8)
Dialysis	111 (15.0)	40 (15.0)
CVVH	114 (15.4)	32 (12.0)

ALL acute lymphocytic leukemia, *AML* acute myeloid leukemia, *BMT* bone marrow transplantation, *CLL* chronic lymphocytic leukemia, *CML* chronic myeloid leukemia, *CVVH* continuous veno-venous hemofiltration, *ED* emergency department, *HSCT* hematopoietic stem-cell transplantation, *ICU* intensive care unit, *IQR* interquartile range, *MDS* myelodysplastic syndrome, *PS* performance status, *SOFA* Sequential Related Organ Failure Assessment
