# Supplementary material for: Direct admission to the intensive care unit from the emergency department and mortality in critically ill hematology patients
Source: Ann Intensive Care. 2019 Oct 2;9:110. doi: 10.1186/s13613-019-0587-7 (PMC6775178; doi:10.1186/s13613-019-0587-7)
Supplement: Supplementary file 3 — Additional file 3. Loveplot. Absolute mean differences in patient characteristics before (unadjusted) and after (adjusted) matching on propensity score. BMT bone marrow transplantation, CLL chronic lymphocytic leukemia, CML chronic myeloid leukemia, HSCT hematopoietic stem-cell transplantation, ICU intensive care unit, MDS myelodysplastic syndrome, PS performance status, SOFA Sequential Related Organ Failure Assessment. [file 13613_2019_587_MOESM3_ESM.pdf]

Additional file 3: Loveplot. Absolute mean differences in patient characteristics before (unadjusted) and after (adjusted) matching on propensity score

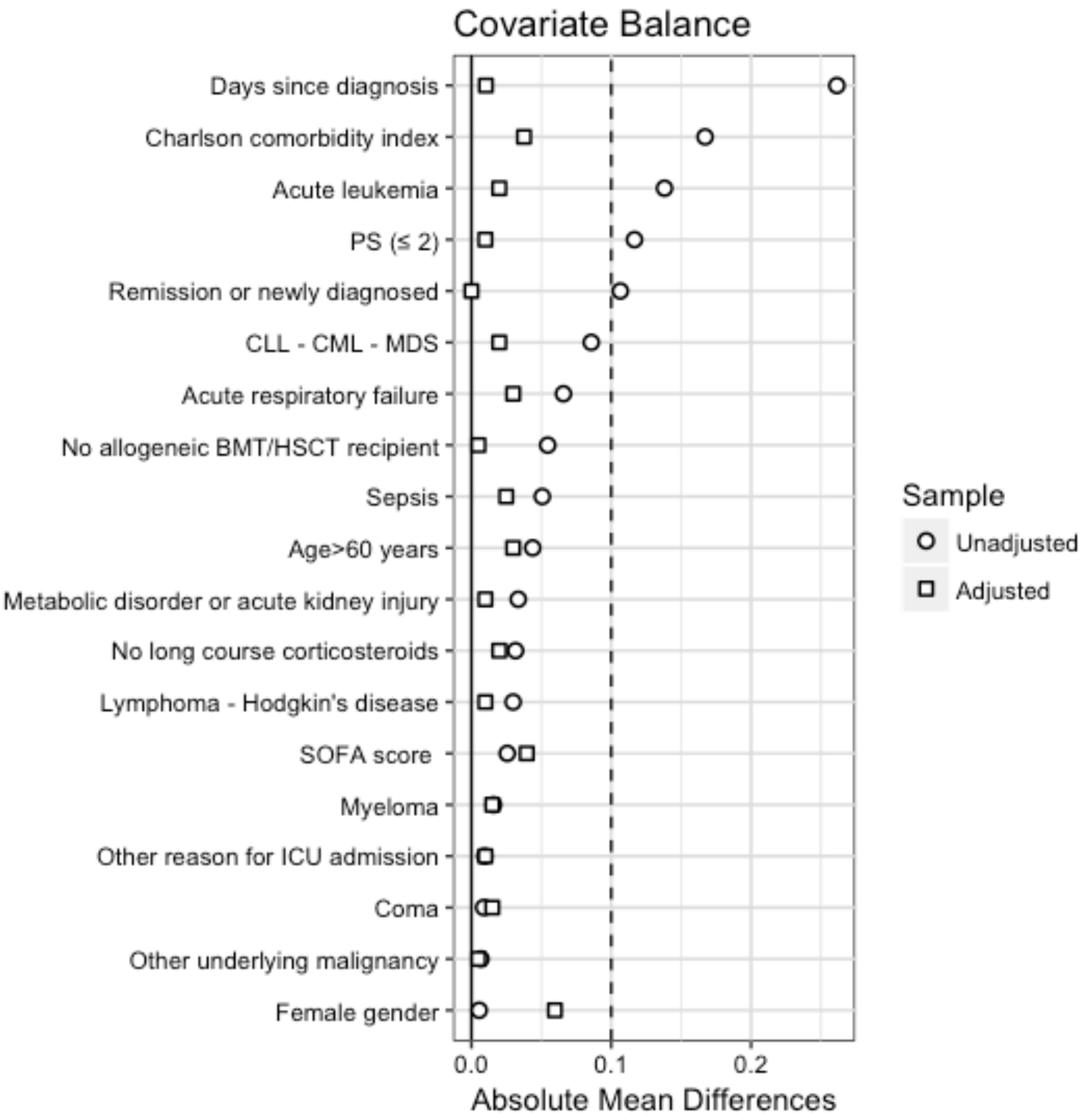

BMT bone marrow transplantation, *CLL* chronic lymphocytic leukemia, *CML* chronic myeloid leukemia, *HSCT* hematopoietic stem-cell transplantation, *ICU* intensive care unit, *MDS* myelodysplastic syndrome, *PS* performance status, *SOFA* Sequential Related Organ Failure Assessment
